# Trials for preparation and evaluation of a combined inactivated reassorted H5N1 and *Escherichia coli* O157 vaccine in poultry

**DOI:** 10.14202/vetworld.2021.1677-1681

**Published:** 2021-06-28

**Authors:** Marwa Fathy El Sayed, Reem A. Soliman, Heba Mohamed Ghanem, Marwa M.S. Khedr, Gina M. Mohamed, Mounir Mohamed Diab El Safty

**Affiliations:** Central Laboratory for Evaluation of Veterinary Biologics, Agricultural Research Center, Giza, Egypt

**Keywords:** avian influenza (H5N1), *Escherichia coli* O157, enzyme-linked immunosorbent assay, hemagglutination inhibition test, Montanide ISA70

## Abstract

**Background and Aim:**

Avian influenza (AI), which is one of the major respiratory diseases of poultry, and *Escherichia coli* (*E. coli*) have caused major economic losses around the world, including in Egypt. Therefore, in this study, we aimed to produce a vaccine from *E. coli* O157 and AI H5N1 formulated with Montanide ISA70 for the protection of poultry against both diseases.

**Materials and Methods:**

We divided one hundred 3-week-old chicks into four groups: Group 1 was vaccinated with prepared inactivated AI H5N1formulated with Montanide ISA70, Group 2 was vaccinated with inactivated *E. coli* formulated with Montanide ISA70, Group 3 was vaccinated with combined *E. coli* and AI H5N1 formulated with Montanide ISA70, and Group 4 was an unvaccinated control group. We measured the immune response using the HI (hemagglutination inhibition) test, enzyme-linked immunosorbent assay (ELISA), and the challenge test.

**Results:**

We found the three vaccines to be safe and sterile during all periods of examination and observation. The HI test showed that Group 1 exhibited specific antibody titers of 2.3 log_2_, 4.3 log_2_, 7.5 log_2_, 7.8 log_2_, 8 log_2_, and 8.1 log_2_ from week 2 to week 7, respectively, post-vaccination. Group 3 exhibited antibody titers of 3.3 log_2_, 5.8 log_2_, 7.8 log_2_, 8 log_2_, 8.3 log_2_, and 8.3 log_2_ from week 2 to week 7, respectively, post-vaccination. The immune response in both groups reached a high titer at week 6. The combined inactivated *E. coli* and AI H5N1 vaccine generated a higher immune response than the inactivated AI H5N1 vaccine, and a significant difference exists between the two groups. For Groups 2 and 3, the ELISA antibody titer exhibited its lowest value, 1996.5 and 2036.7, respectively, at week 1 post-vaccination; whereas, both groups exhibited the highest titers, 2227.7 (for Group 2) and 2287.3 (for Group 3), in week 3 post-booster. The ELISA for the combined inactivated *E. coli* and AI H5N1 vaccine had a higher titer than did the inactivated *E. coli* vaccine, and a significant difference exists between the two groups. Moreover, the protection rate was higher in Group 3, with 100% for *E. coli* and 90% for the AI H5N1 vaccine.

**Conclusion:**

Our findings demonstrate that producing a combined vaccine using *E. coli* and AI H5N1 formulated with Montanide ISA70 is recommended for protection against both diseases.

## Introduction

Avian influenza (AI) is a poultry disease with the potential to cause major epidemics resulting in significant economic losses. AI is an economically important respiratory disease for poultry in Egypt and across the globe. AI viruses belong to type A of the *Orthomyxoviridae* family, and 18 hemagglutinins (H1–H18) and 11 neuraminidase subtypes (N1–N11) have been reported.

One possible explanation for such a high mortality rate and great financial losses can be a mixed infection with other respiratory pathogens. A member of *Enterobacteriaceae* family, pathogenic *Escherichia coli* (*E. coli*) O157 is a factor that can affect the pathogenicity of the AI H5N1 virus [1]. This bacterium is categorized on the basis of its somatic and flagellar antigens. Colisepticemia or aerosol disease is the most important disease in poultry caused by avian pathogenic *E. coli* (APEC) strains resulting in high mortality, and it is the most common bacterial infection in poultry. This infection usually occurs among birds 2-12 weeks of age, with the majority of the cases occurring among birds 4-9 weeks of age, with mortality rates as high as 20%.

In broilers, colibacillosis is often considered secondary to other pathogens and unfavorable environmental conditions such as the Newcastle disease virus, the infectious bronchitis virus, and the AI virus [2]. The combination of bacterial and viral vaccines containing multiple antigens has many benefits for manufacturers, as it reduces production costs; for administrators, as it saves time and efforts as well as simplifies the immunization schedule; and for the animals, as it minimizes the stress of multiple vaccinations [3].

A recent study in Egypt by Marwa *et al*. [4] who isolated and identified a new strain of *E. coli* O175, which caused severe economic losses in several poultry farms in Egypt along with AI, and by Azza *et al*. [5], who detected the pathogenicity of *E. coli* serogroup O157 in broiler chicks, indicating a threat not only to the birds but also to public health. Therefore, we have focused our study on this topic.

Therefore, in this study, we aimed to produce a vaccine from *E. coli* O157 and AI H5N1 formulated with Montanide ISA70 for the protection of poultry against both diseases.

## Materials and Methods

### Ethical approval

The study was approved by Institutional Animal Care and Use Committee at Central Laboratory for Evaluation of Veterinary Biologics, Cairo, Giza, Egypt.

### Study period and location

The study was conducted in March 2020 at Central Laboratory for Evaluation of Veterinary Biologics, Cairo, Giza, Egypt. 

### Strains used

*E. coli* serotype O157, which was isolated locally [4], was inoculated on MacConkey agar medium and incubated at 37°C for 24 h. The strain was identified biochemically using the API 20E identification system following the procedures in the Biomerieux kit manual. The AI virus (HPAI/chicken/Egypt/D10552B/2015 H5N1), obtained from the National Research Center, Giza, Egypt, was egg adapted with titer.

### Chickens

A total of one hundred and sixty 3-week-old specific pathogen-free (SPF) chicks were obtained from Koom-Osheim Farm in the Faiyum Governorate. The chickens were fed *ad libitum* without any antibacterial or anticoccidial components in their feed.

### Vaccine preparation

*E. coli* O157 was grown separately on brain heart agar and incubated at 37°C for 24 h. The colonies were collected using normal saline and mixed, and the bacterial suspension was adjusted to be 1×10^9^ CFU/0.5 mL (vaccinal dose). The bacteria were inactivated by adding 0.5% formalin with agitation, then Montanide ISA70 (SEPPIC^®^, France) was mixed with one part of the bacterial suspension in a ratio of 70:30 (w/w) (adjuvant: antigen) [6].

Propagation and titration of AI H5N1, which was isolated locally, were done in embryonated chicken egg SPF that was 9-10 days old, according to Chaffer *et al*. [6]. Its titer was 10^9^ EID50/mL. Inactivation of the AI virus was carried out using binary ethylenimine (0.1 M), with a final concentration of 0.01 M. Inactivation and its testing were done according to Sarachai *et al*. [7].

A combined vaccine of *E. coli* and AI H5N1 was prepared in water emulsion by mixing equal volumes of the inactivated *E. coli* antigenic phase and inactivated AI antigenic phase to form an aqueous phase and then was mixed with Montanide ISA70 in a ratio of 70:30 (w/w) (adjuvant: antigen).

### Experimental design

A total of one hundred and sixty 3-week-old SPF chicks were divided into four groups, with 40 in each:

- For Group 1, chicks were vaccinated with inactivated AI H5N1 formulated with Montanide ISA70 in a dose of 0.5 mL S/C (1×10^9^ CFU/dose)
- For Group 2, chicks were vaccinated with inactivated *E. coli* formulated with Montanide ISA70 using the same dose as Group 1
- For Group 3, chicks were vaccinated with combined inactivated *E. coli* and AI H5N1 formulated with Montanide ISA70 in a dose of 0.5 mL S/C
- For Group 4, chicks were injected with 0.5 mL S/C normal saline; this was the control group.

Birds in Groups 2 and 3 were given booster dose of the vaccine (*E. coli* only) through the same route and in the same dose, 3 weeks after the first immunization. Serum samples were obtained regularly before immunization, weekly after each vaccination, and post-challenge for 2 weeks (once/week). These serum samples were then pooled and stored at −20°C until use during follow-up for the induced antibodies.

### Quality control testing of the prepared experimental vaccines

The vaccines were tested for sterility and safety following the standard international protocol [8] before usage in the field trial.

### Determination of an immune response to the prepared experimental vaccines

The enzyme-linked immunosorbent assay (ELISA) test was performed on the serum sample of tested chickens according to the method described by Voller *et al*. [9] and Briggs and Skeels [10], for evaluation of immune response against *E. coli* in Groups 2 and 3. The results were calculated according to the following formula:


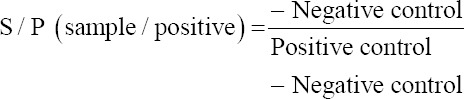


- Log10 titer = 1.09 (log10 S/P) + 3.63

- Titer = Antilog 10.

The HI (hemagglutination inhibition) test was carried out [8] for the evaluation of immune response in Groups 1 and 3 against AI.

For the challenge tests for the virulent *E. coli* strains, 3 weeks after the booster dose, vaccinated chickens (20 birds/flock) from Groups 2 and 3 as well as the non-vaccinated birds were injected in the thigh region with 0.2 mL (containing 10^7^ CFU) of *E. coli* serotype O157 and monitored for clinical signs. Mortality was recorded for 7 days after the challenge according to the method described by Chaffer *et al*. [6].

For the challenge tests for the AI H5N1 strain, 20 vaccinated chickens from Groups 1 and 3, as well as 10 non-vaccinated birds, were challenged by inoculation of 0.1 mL of AI H5N1 (10^6^ EID50) strain through the intranasal route 4 weeks after first and a booster dose of vaccination. Then, tracheal and cloacal swabs were collected on days 1, 3, 5, 7, and 10 post-challenge to check for virus shedding. Mortality was recorded to calculate the percentage of protection [11].

### Statistical analysis

We analyzed the results of the HI tests (Table-1) and ELISA tests (Table-2) and compared the parametrical correlations using Student’s t-test [12]**.** Significance level was set at p>0.05

**Table-1:** Mean AI-HI antibody titer in vaccinated chickens with inactivated monovalent AI vaccine (Group 1) and bivalent (*E*. *coli* and AI) vaccine (Group 3).

Weeks	Mean AI-HI antibody titers*		

	Group 1	Group 3*	Group 4
1^st^	0	0	0
2^nd^	2.3	3.3	0
3^rd^	4.3	5.8	0
4^th^	7.5	7.8	0
5^th^	8.5	9	0
6^th^	9.6	10.8	0
7^th^	10.7	11	0

Group 1: Vaccinated with inactivated AI vaccine formulated with Montanide ISA70. Group 3: Vaccinated with combined inactivated *E. coli* +AI vaccine formulated with Montanide ISA70. Group 4: Control group.

* Antibody titers were expressed as log2. *Significant at p>0.05. AI=Avian influenza, E. *coli*=*Escherichia coli*

**Table-2:** ELISA antibody titer against *E. coli* O157 in vaccinated chicken.

Groups	ELISA antibody titer										

	Pre-vaccination	Weeks post-vaccination			Booster	Weeks post-boostering			Challenge#	Weeks post-challenge	
		
		1	2	3		1	2	3		1	2
Group (2)	93	1996.5	2151.6	2172.2		2025.8	2192.5	2227.7		2100.5	2254.1
Group (3)*	110	2036.7	2046.8	2166.1		2171.8	2202.1	2287.3		2061.2	2469.3
Control	110	193	120	244		157	166	244		120	106

Group (2): Vaccinated with inactivated *E. coli* vaccine formulated with Montanide ISA70. Group (3): Vaccinated with combined inactivated E. coli +AI vaccine formulated with Montanide ISA70. #Challenge with virulent *E. coli* (O157) strain. *Significant at p>0.05. AI=Avian influenza, *E. coli*=*Escherichia coli*, ELISA=Enzyme-linked immunosorbent assay

## Results

**Figure-1:**
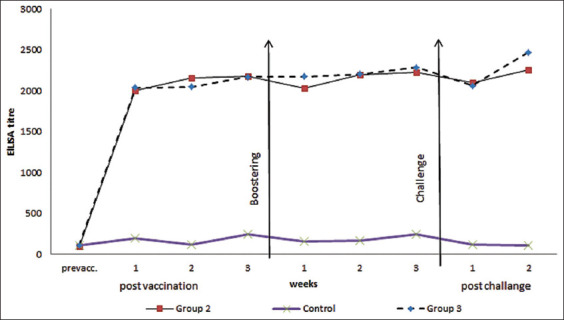
Enzyme-linked immunosorbent assay antibody titer against Escherichia coli O157 in vaccinated chicken.

We found all three vaccines to be safe and sterile during all periods of examination and observation. The humoral immune response against the reassorted AI H5N1 measured by the HI test is illustrated in Table-1, while the immune response against *E. coli* serotype O157 measured by ELISA is illustrated in Table-2 and Figure-1.

### HI tests

The HI tests for the vaccinated chickens showed that Group 1 exhibited specific antibody titers of 2.3 log_2_, 4.3 log_2_, 7.5 log_2_, 7.8 log_2_, 8 log_2_, and 8.1 log_2_ from week 2 to week 7, respectively, post-vaccination (Table-1). Group 3 exhibited antibody titers of 3.3 log_2_, 5.8 log_2_, 7.8 log_2_, 8 log_2_, 8.3 log_2_, and 8.3 log_2_ from week 2 to week 7, respectively, post-vaccination. The immune response in both groups reached a high titer at week 6. Thus, the combined inactivated *E. coli* and AI vaccine gives a higher immune response than the inactivated AI vaccine, with a significant (p>0.05) difference between the two groups.

### ELISA

In the ELISA tests, antibody titer against *E. coli* O157 in vaccinated chickens is shown in Table-2 and Figure-1. In Groups 2 and 3, the ELISA antibody titer exhibited the lowest values–1996.5 and 2036.7, respectively at 1 week post-vaccination; whereas, both groups exhibited the highest titer–2227.7 for Group 2 and 2287.3 for Group 3–at 3 week after the booster dose (Table-2 and Figure-1). Thus, the combined inactivated *E. coli* and AI vaccine gave a higher titer than the inactivated *E. coli* vaccine, with a significant (p>0.05) difference between the two groups.

### Challenge tests

In the AI challenge tests, Group 1 had 80% protection rate and Group 3 had 90% protection rate (Table-3). The unvaccinated control Group 4 was unable to withstand the virulent challenge virus, with 0% protection. In *E. coli* challenge tests, Group 2 had 90% protection rate and Group 3 had 100% protection rate (Table-4). Control Group 4 had 0% protection.

**Table-3:** Results of challenge test among chickens vaccinated with inactivated AI vaccine and combined inactivated *E. coli* and AI vaccine against serotype (H5N1).

Groups of chicken	Total no. of challenged birds	Days post-challenge										Total death	Protection rate	Reisolation

		1	2	3	4	5	6	7	8	9	10			
Group (1)	20	-	1	3								4/20	80%	−ve
Group (3)	20	-	-	1	1							2/20	90%	−ve
Control	10	3	2	5								10/10	0%	+ve

AI=Avian influenza, *E. coli=Escherichia coli*

**Table-4:** Results of challenge test among chickens vaccinated with inactivated *E. coli* vaccine and combined inactivated *E. coli* and AI vaccine against *E. coli* serotype O157.

Groups of chicken	Total no. of challenged birds	Days post-challenge										Total death	Protection rate	Reisolation

		1	2	3	4	5	6	7	8	9	10			
Group (2)	20	-	1	1								2/20	90%	−ve
Group (3)	20	-	-		\						0/20	100%	−ve
Control	10	3	2	5								10/10	0%	+ve

AI=Avian influenza, *E. coli=Escherichia coli*

## Discussion

Bacteria such as *E. coli* and viral diseases like AI represent major problems in the poultry industry. APEC causes a systemic disease that is highly lethal in both broiler and layer chickens and is the leading cause of the 1^st^ week mortality in layers [13]. AI H5N1 is a highly contagious viral disease, which affects several species of birds, and is caused by influenza virus type A, which is a member of the family *Orthomyxoviridae* [14].

Vaccination is the cornerstone in controlling and eradicating such diseases. One previous study has shown that inactivated vaccines are capable of increasing antibody response, which protect the birds from death and against a decline in egg production [15]. Vaccine quality improvement is also necessary. An effective vaccine needs not only a good antigen but also a preferable adjuvant to enhance both cellular and humoral immunities. In addition, reducing post-vaccination side effects using mineral oil emulsion vaccine potentiates the antibody response and prolongs the duration of immunity [16]. Montanide ISA70 oil adjuvant, which is characterized by low viscosity and easy injectability, also enhances the cellular and humoral immunities, increasing the protection limit of the vaccine [17].

The vaccines tested by us were found to be safe, protecting the chickens without any untoward reactions. Using Montanide ISA70 provided a high immune response and protection rate in the three vaccines; the combined bivalent vaccine gave higher titer and protection than the monovalent vaccine. This finding agrees with the study of El-Sayed *et al*. [17] who revealed that Montanide ISA206 or ISA70 oil adjuvants confer good, long-lasting protection. Our results of the ELISA tests were in agreement with those of Mohammed *et al*. [18] who found that the ELISA titer against *E. coli* for an inactivated vaccine with Montanide ISA70 was higher than inactivated vaccine with aluminum hydroxide gel as an adjuvant.

In our experimental trials, the chickens vaccinated with combined inactivated *E. coli* and AI gave a higher immune response at week 7 than those vaccinated with the inactivated *E. coli*. In an investigation of HI and ELISA tests of the sera of chickens vaccinated with combined AI (H9N2) and fowl cholera (FC), the study by Salama *et al*. [19] indicated similar results until week 6 post-vaccination for FC using Montanide ISA206. In addition, Ghanem *et al*. [20] have stated that using Montanide ISA70 as an adjuvant in preparation of bivalent combined vaccine (AI + FC), monovalent AI vaccine and monovalent FC vaccine gave better post-vaccine reaction and higher immune response than using white oil adjuvant.

The two vaccines in Groups 1-3 had protection rates of 80-100%. These results agree with those of Salama *et al*. [19] who found protection levels of 80-93.3% against *Pasteurella multocida* and 96.6% against AI virus after challenging with the used virulent strains after the single and booster doses, respectively. Moreover, Ghanem *et al*. [20] found a high degree of protection for layers and chicks with a vaccine prepared from trivalent *E. coli* (serotypes O1, O2, and O78) and an inactivated Newcastle disease. Further, Erganis *et al*. [21] have declared that both the heat and formalin-inactivated aluminum precipitated vaccines prepared with the virulent *E. coli* isolates were effective in protecting chickens of different ages against various forms of avian colibacillosis.

## Conclusion

The chickens in our study, which were vaccinated with inactivated *E. coli*, AI H5N1, and both combined, exhibited a high immune response and rate of protection, thereby suggesting the development of strong and long-lasting immunity. Thus, we concluded that producing a vaccine from *E. coli* and AI H5N1 formulated with Montanide ISA70 can protect against both *E. coli* and AI, helping to mitigate the economic impact of these diseases.

## Authors’ Contributions

All authors designed and planned this research work. MMSK and HMG: Identified the strains. RAS and GMM: Prepared the vaccine. MFE and RAS: Experimental design and challenge test were carried out. MFE, RAS, HMG, MMSK, and GMM: Determination of immune response. MMDE: Statistical analysis. All authors contributed equally in preparation and revision of the manuscript and collection of scientific papers related to the subject of this research. All authors read and approved the final manuscript.
